# Safety and immunogenicity of inactivated COVID-19 vaccine CoronaVac and the RBD-dimer–based COVID-19 vaccine ZF2001 in chronic hepatitis B patients

**DOI:** 10.3389/fmed.2023.1078666

**Published:** 2023-02-08

**Authors:** Shiheng Wu, Xiaolin Wang, Mingyang Feng, Xiaoman Liu, Xinxing Fan, Xiangui Ran, Baogui Wang, Hui Wang

**Affiliations:** ^1^Department of Infectious Diseases, Fuyang People's Hospital, Fuyang, China; ^2^Department of Infectious Diseases, Ruijin Hospital, Shanghai Jiao Tong University School of Medicine, Shanghai, China; ^3^Department of Respiratory and Critical Care Medicine, Fuyang People's Hospital, Fuyang, China

**Keywords:** COVID-19 vaccine, CHB patients, safety, immunogenicity, ZF2001, CoronaVac

## Abstract

**Background and aims:**

Although COVID-19 vaccination is recommended for the patients with chronic liver disease, the clinical outcomes of COVID-19 vaccinated in patients with chronic hepatitis B (CHB) has not been well characterized. The study aimed to explore the safety and specific antibody responses following COVID-19 vaccination among CHB patients.

**Methods:**

Patients with CHB were included. All patients were vaccinated with two doses of inactivated vaccine (CoronaVac) or three doses of adjuvanted protein subunit vaccine (ZF2001). The adverse events were recorded and neutralizing antibody (NAb) were determined 14 days following the whole-course vaccination.

**Results:**

A total of 200 patients with CHB were included. Specific NAb against SARS-CoV-2 were positive in 170 (84.6%) patients. The median (IQR) concentrations of NAb were 16.32 (8.44–34.10) AU/ml. Comparison of immune responses between CoronaVac and ZF2001 vaccines showed no significant differences in neither the concentrations of NAb nor the seropositive rates (84.4 vs. 85.7%). Moreover, we observed lower immunogenicity in older patients and in patients with cirrhosis or underlying comorbidities. The incidences of adverse events were 37 (18.5%) with the most common adverse event as injection side pain [25 (12.5%)], followed by fatigue [15 (7.5%)]. There were no differences in the frequencies of adverse between CoronaVac and ZF2001 (19.3% vs. 17.6%). Almost all of the adverse reactions were mild and self-resolved within a few days after vaccination. Severe adverse events were not observed.

**Conclusions:**

COVID-19 vaccines, CoronaVac and ZF2001 had a favorable safety profile and induced efficient immune response in patients with CHB.

## Introduction

Following the pandemic of COVID-19, it is the most priority to control transmission of SARS-CoV-2 (1). Previous studies report that the patients with chronic liver disease, particularly cirrhosis presented worsened outcomes following COVID-19 infection (2, 3), presenting more disturbed liver abnormalities (4) and could lead to hepatitis B reactivation which can cause liver failure (5). Therefore, liver societies have recommended vaccination against COVID-19 for all patients with chronic liver diseases (6–8).

Although remarkable progress has been made in developing vaccines, only a few participants with pre-existing chronic liver diseases were included in clinical trials studying the safety and efficacy of COVID-19 vaccines. Recent studies have reported the responses of COVID-19 mRNA or inactivated vaccines in patients with nonalcoholic fatty liver disease (9), liver transplant recipients (10) and in chronic hepatitis B (CHB) patients (11). However, there is no detailed information available on the use of the SARS-CoV-2 adjuvanted protein subunit vaccine (ZF2001) (12) in CHB patients. In addition, the safety and effectiveness of ZF2001 vaccine remain to be clarified (13).

Given the large number of chronic hepatitis B patients in China (14), we aimed to explore the safety and immunogenicity of COVID-19 vaccines (CoronaVac and ZF2001) in CHB patients in this prospective study.

## Methods

### Study design

The study was performed at Fuyang People's Hospital in Anhui, China. Among 200 recruited CHB patients, 109 were vaccinated with inactivated virus vaccine against SAR-CoV-2 (CoronaVac) and 91 were vaccinated with adjuvanted protein subunit vaccine (ZF2001). The vaccination regimen for CoronaVac is two doses (3 ug) given intramuscularly with an interval of 3 weeks. The vaccination regimen for ZF2001 is a total of three doses (25 μg) given intramuscularly with an interval of >4weeks. The diagnostic criteria for CHB infection are: HBsAg or HBV DNA positive for at least 6 months. Exclusion criteria were: co-infection of HBV and HIV, HCV, HDV, EBV, or CMV, evidence of schistosomiasis or Wilson's disease, received antiviral therapy, alcohol liver disease (alcohol consumption ≥40 g/day for male and ≥20 g/day for female), or liver damage induced by other causes (non-alcoholic fatty liver, drugs, autoimmune hepatitis). All participants were over the age of 18 and had no known history of SARS-CoV-2 infection. Clinical data on anti-HBV therapy, HBV serological biomarkers, and liver function test results were extracted from electronic medical records prior to the first vaccination. Abnormal ALT test was defined as a value greater than the upper limit of normal (F: 40 U/L, M: 50 U/L). The presence or absence of liver cirrhosis is determined based on clinical evidence combined with liver imaging examinations. The current study is approved by the Ethics Committee of Fuyang People's Hospital. Under the guidance of professional physicians, adverse reactions after vaccination, including local (pain, swelling, induration) or systemic reactions (fever, fatigue, drowsiness, headache, dizziness, myalgia), were collected by filling out a standard questionnaire. The primary safety outcome was the overall incidence of adverse events within 7 days. The study was approved by the ethics committees of Fuyang People's Hospital.

### Anti-SARS-CoV-2 NAb measurement

Plasma samples were collected 2 weeks following vaccination, and neutralizing antibodies (NAbs) were detected, using the SARS-CoV-2 Neutralizing Antibody Kit (CLIA) (China-based Maccura Biotechnology Co., Ltd). Concentrations equal to or higher than 6 AU/ml are considered positive immune responses, according to the Neutralizing Antibody Kit instructions.

### Statistical analysis

Continuous variables were presented as mean ± standard deviation and categorical variables were presented as *n* (%). The concentrations of NAb were presented as median with interquartile range (IQR). GraphPad Prism v8.0 was used for statistical analysis. Two groups' continuous variables comparison were analyzed with Student *t*-test or Mann-Whitney U test. The categorical variables were analyzed with χ2 test. For all tests, a two tailed *p* < 0.05 was considered statistically significant.

## Results

### Participant's characteristics

Total of 200 patients with pre-existing CHB were eligible for analysis. The average age was 47.39 ± 13.60 years and 108 (54.0) were male. The mean BMI was 24.29 ± 2.62 kg/m (2). Among these CHB patients, 12 (6%) were diagnosed as CHB-related liver cirrhosis. Comorbidities were presented in 24 (12%) CHB patients with hypertension as the most prevalent condition (6%), followed by fatty liver (3.5%), diabetes (3%), hyperlipidemia (1%), coronary artery disease (1%), cerebrovascular disease (1%), and chronic kidney disease (0.5%) (Table 1).

**Table 1:** Baseline characteristics of study cohort.

**Characteristics**	**Patients (*n* = 200)**
Age, (mean ± SD), years	47.39 ± 13.60
Age groups, *n* (%)	
20–35, *n* (%)	43 (21.5)
35–50, *n* (%)	74 (37.0)
50–65, *n* (%)	57 (28.5)
65–80, *n* (%)	26 (13.0)
Gender	
Female, *n* (%)	92 (46.0)
Male, *n* (%)	108 (54.0)
Body mass index, mean ± SD, kg/m^2^	24.29 ± 2.62
Cirrhosis, *n* (%)	12 (6.0)
Any comorbidity, *n* (%)	24 (12)
Hypertension, *n* (%),	12 (6.0)
Fatty liver, *n* (%)	7 (3.5)
Diabetes, *n* (%)	6 (3.0)
Hyperlipidemia, *n* (%)	2 (1.0)
Coronary artery disease, *n* (%)	2 (1.0)
Cerebrovascular disease, *n* (%)	2 (1.0)
Chronic kidney disease, *n* (%)	1 (0.5)
Others, *n* (%)	2 (1.0)

### Vaccine safety

Among the 200 patients, 37 (18.5) patients reported adverse effects after the vaccination. Injection-site pain was the most frequent local adverse event (12.5%), followed by induration (2.0%) and itch (1.0%). The most common systemic adverse event was fatigue (7.5%), followed by drowsiness (2.5%), fever (2.5%), nausea (0.5%) and abdominal bloating (0.5%). Almost all of the adverse reactions were mild and self-resolved within a few days after vaccination. Serious side effects were not observed (Table 2). Importantly, the frequencies of adverse events from the CHB patients with cirrhosis or comorbidities were similar to the patients without them, suggesting the safety of COVID-19 vaccination in CHB patients regardless of cirrhosis or comorbidities (Supplementary Figures 2A, B). There were no differences in the frequencies of adverse events in CHB patients receiving different types of vaccines (19.3 vs. 17.6%) (Supplementary Figure 2C).

**Table 2:** Safety and immunogenicity of COVID-19 vaccination in patient with CHB.

**Characteristics**	**Patients (*n* = 200)**
Total reactions within 7 days after each injection	
Any, *n* (%)	37 (18.5)
Injection site adverse reactions	
Pain, *n* (%)	25 (12.5)
Induration, *n* (%)	4 (2.0)
Itch, *n* (%)	2 (1.0)
Systemic adverse reactions	
Fatigue, *n* (%)	15 (7.5)
Drowsiness, *n* (%)	5 (2.5)
Fever, *n* (%)	5 (2.5)
Nausea, *n* (%)	1 (0.5)
Abdominal bloating, *n* (%)	1 (0.5)
Antibody responses after whole-course vaccination	
Neutralizing antibody, median (IQR), AU/ml	16.32 (8.44–34.10)
Neutralizing antibody response, *n* (%)	170 (84.6)
IgM positive, *n* (%)	16 (8.0)
IgG positive, *n* (%)	173 (86.1)

**Figure 1:**
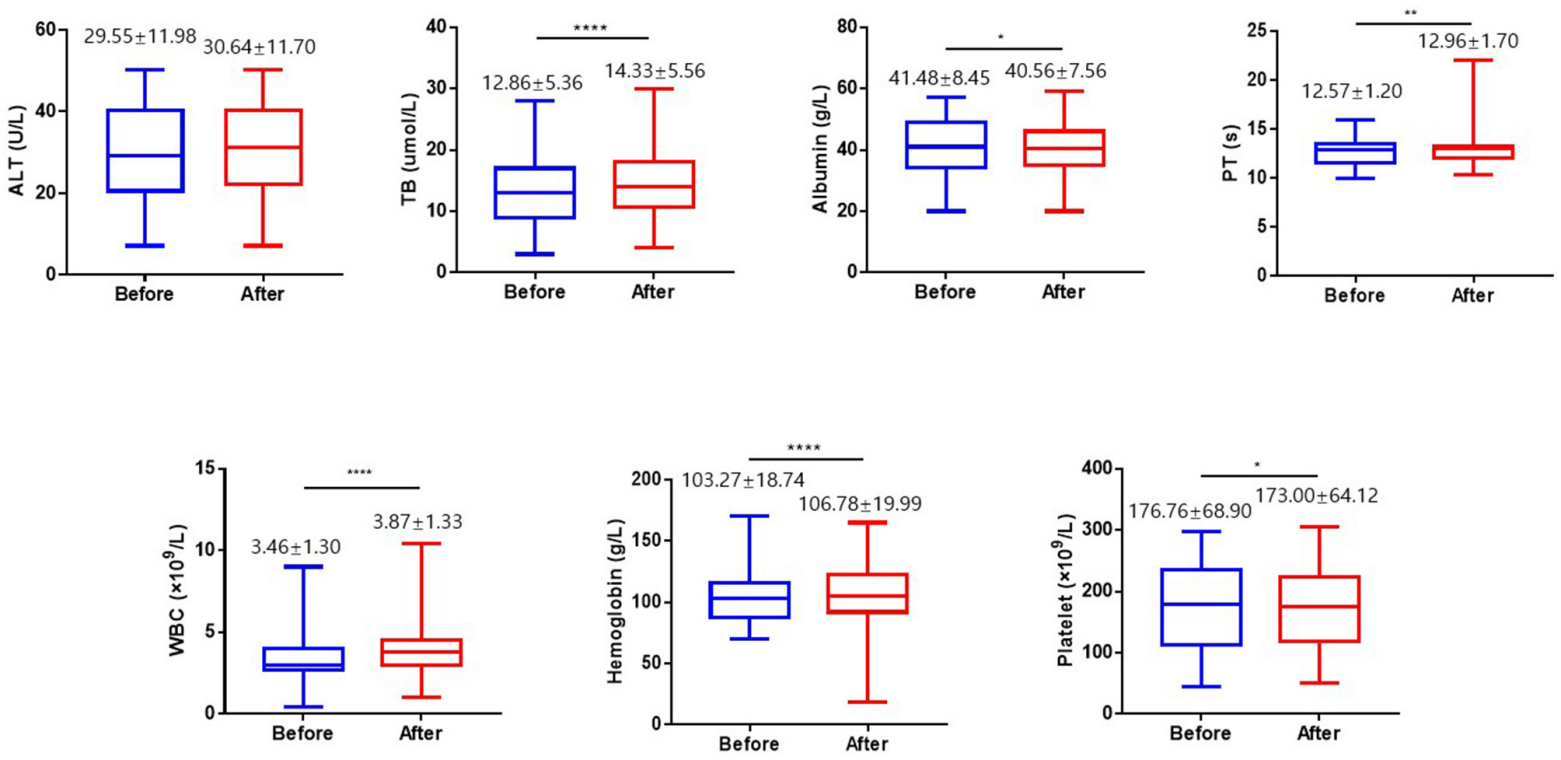
The biochemical characteristics before and after vaccination. The serum levels of alanine amino transferase (ALT), total bilirubin (TB), and albumin were compared before and after vaccination. The value of prothrombin time (PT), the levels of white blood cell, hemoglobin and platelet in the blood were compared before and after vaccination. ALT, alanine amino transferase; TB, total bilirubin; PT, prothrombin time; WBC, white blood cell. *p < 0.05, **p < 0.01, ****p < 0.0001.

To further understand the safety of vaccines in CHB patients, we compared the biochemical characteristics prior to and post vaccination. Total bilirubin (TB), prothrombin time (PT), white blood cell (WBC) and hemoglobin levels were increased by 1.11, 1.03, 1.12 and 1.03 fold, respectively (*p* < 0.05) in trend after vaccination and albumin and platelet levels were decreased by 0.98 fold (*p* < 0.05) (Figure 1). Although these basal biochemical characteristics were changed, they were all within the normal range. Thus, we believe that COVID-19 vaccines appeared to be safe in CHB patients.

### Neutralizing antibody (NAb) titers

**Figure 2:**
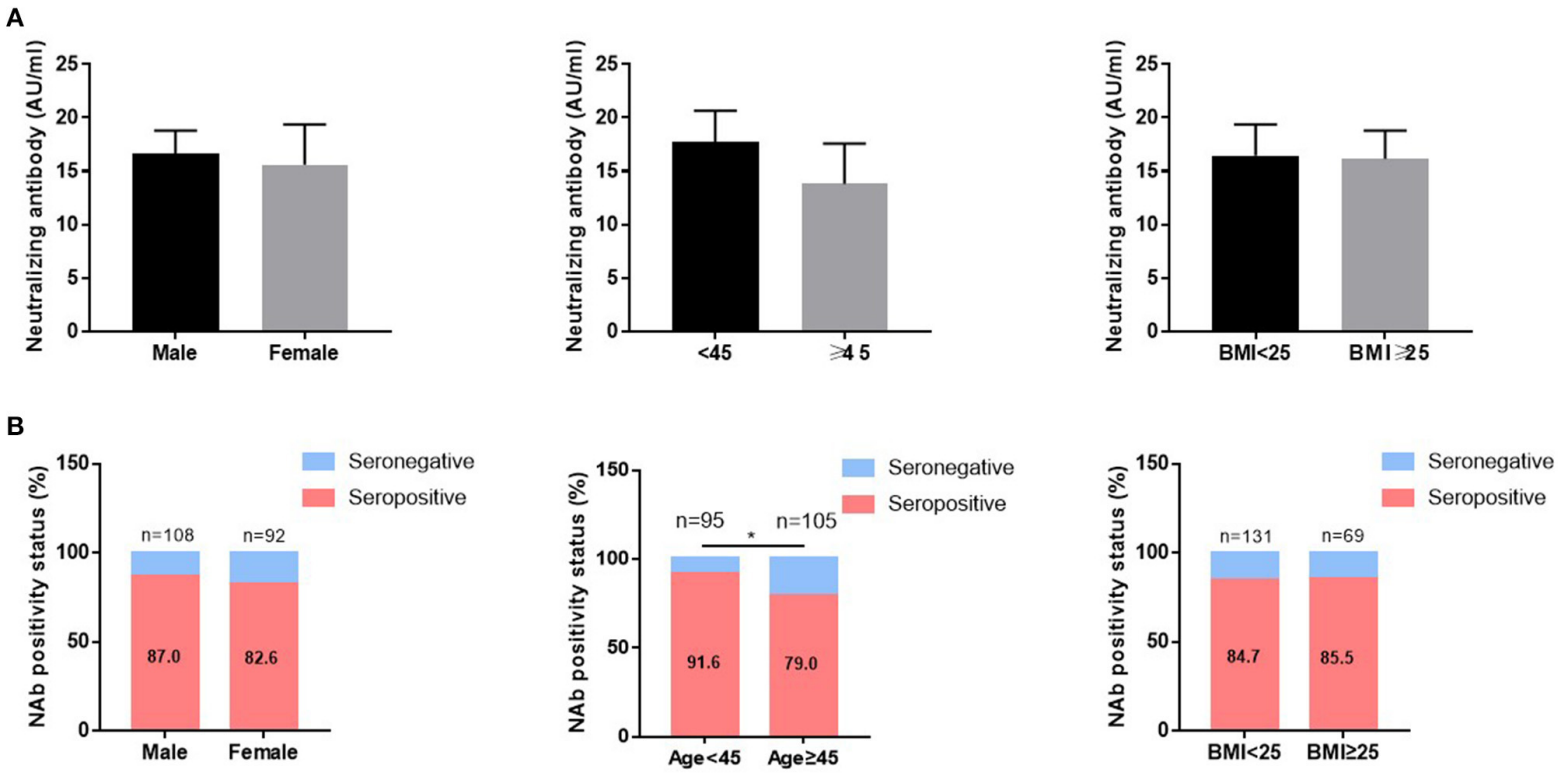
The influence of age, sex, and BMI on the immunogenicity of vaccines in CHB patients. The neutralizing antibody (NAb) concentrations **(A)** and NAb positive rates **(B)** were compared according to sex, age, and BMI in patients with chronic hepatitis B. ***P < 0.0001.

The median (IQR) concentrations of NAb were 16.32 (8.44–34.10) AU/ml. The overall NAb response rates were 84.6%. Furthermore, IgM or IgG antibodies were present in 16 (8%) or 173 (86.1%) CHB patients 14 days post vaccination (Table 2). The concentrations of NAb were further stratified according to sex, age, and BMI, no significant differences were observed (Figure 2A). However, the positive rates of immune responses were significantly higher in younger CHB patients (< 45 yrs) compared to older CHB (≥45 yrs) (91.6 vs. 79.0%, *p* < 0.05) (Figure 2B, middle panel). Comparison of immune responses between CoronaVac and ZF2001 vaccines showed no significant differences in neither the concentrations of NAb (Supplementary Figure 1A), nor the seropositive rates (84.4 vs. 85.7%) (Supplementary Figure 1B), suggesting that the immunogenicity was comparable between CoronaVac and ZF2001 vaccines.

Finally, to determine whether comorbidities affect the immune responses of COVID-19 vaccine, we compared the concentrations of NAb and seropositive rates in CHB patients with and without comorbidities. The concentrations NAb and seropositive rates were lower in the CHB patients with comorbidities compared to the CHB patients without comorbidities (Supplementary Figures 1C, D). In addition, among 30 CHB patients who had no response to vaccines, eight of them had CHB-related cirrhosis. Then we compared the immune responses in CHB patients with and without cirrhosis. The concentrations of NAb were dramatically low in patients with cirrhosis compared to patients without cirrhosis [5.18 (3.95–11.82) vs 17.04 (8.79–34.41)] (Supplementary Figure 1E) and seropositive rates were also lower in patients with cirrhosis compared to patients without cirrhosis (33.3 vs. 88.3%) (Supplementary Figure 1F).

## Discussion

In our current study, 37 (18.5%) CHB patients reported adverse effects post the COVID-19 vaccination. The incidence of adverse effects in our study was similar to the adverse effects in volunteers (18.9%) post CoronaVac vaccination in Turkey (15), while it was lower than the overall incidence of adverse reactions (30.2%) in CHB patients after receiving inactivated vaccines (BBIBP-CorV, CoronaVac, or WIBP-CorV) (11), and lower than the incidence of adverse effects in NAFLD patients after BBIBP-CorV vaccination (29.4%) (9). There was only slight but not significant change of biochemical characteristics prior to and post vaccination. Taken together, our results support that CHB patients are safely vaccinated with COVID-19 vaccines.

To investigate the efficiency of two types of COVID-19 vaccine, CoronaVac and ZF2001 in CHB patients, NAbs were measured. Studies have showed that compared to the wild type, Omicron variant possesses comparable binding affinity to human ACE2 in comparison with the wild type SARS-CoV-2, and Delta variant possesses stronger binding affinity to human ACE2 than Omicron variant (16). Thus, neutralizing antibodies directed against both the original strains and the mutant strains of Wuhan. In several clinical trials and studies for CoronaVac and ZF2001, to evaluate the immunogenicity of the vaccine, the neutralizing antibodies were analyzed 14 days or 9–21 days after receiving the last dose of vaccination (17, 18). Thus, it is acceptable to analyze neutralizing antibodies 14 days following the whole-course vaccination in our study. The positivity rate of NAb was 84.6% in our study, which is consistent with previous report in non-CHB cohort (11), suggesting that COVID-19 vaccinations are effective, regardless of CHB status. In addition, we found that older CHB patients exhibited weaker humoral immunity to vaccination than younger ones, suggesting that age is a contributing factor in determining host immunity. This is in line with other studies which also indicated that seropositivity decreased with increasing age (15, 19).

Cirrhosis contributes to deregulated immunity in the host (20). We revealed that the concentrations of NAb and seropositive rates were dramatically lower in patients with cirrhosis, compared to the patients without cirrhosis. These results were in line with previous study indicating that cirrhosis is associated with poor antibody response in patients with chronic liver diseases (21). Due to the limited number of patients with cirrhosis (*n* = 12), we cannot generally conclude that immunogenicity of COVID-19 vaccination was less effective in patients with cirrhosis. Further studies are needed in a larger group of patients.

In our study, 12% patients had underlying comorbidities. We observed lower efficiency of vaccine in patients with comorbidities, as evidenced by the decreased NAb concentrations and NAb seropositive rates in patients with comorbidities compared to patients without comorbidities. Unfortunately, due to the small number of patients we studied, it is difficult to find out which morbidities are responsible for the attenuated immune response. Previous studies have shown that the presence of underlying comorbidities, such as hypertension, diabetes, cardiovascular disease, and cerebrovascular disease are risk factors for COVID-19 infection (22) and lead to poor prognosis (23). Despite the possibility of reduced immune response, the benefits of the COVID-19 vaccination outweigh the risks. Our study support the recommendations that suggest COVID-19 vaccination for patients with comorbidities (24).

We realize that there are some limitations in the current study: the number of CHB patients, especially patients with CHB-related cirrhosis were relatively small, thus it is difficult to come to a convincing conclusion about the immunogenicity of COVID-19 vaccine in CHB patients with cirrhosis. Secondly, the clinical stages of HBV infection of CHB patients were not recorded. It will be interesting to analyze the antibody responses after COVID-19 vaccination in CHB patients with different immune phases. Thirdly, anti-viral therapy was not recorded in our study. In addition, we did not include normal people with negative hepatitis B, as control in this study. Further studies might be carried out to analyze the impacts of vaccination on antiviral therapy in CHB patients.

## Data availability statement

The raw data supporting the conclusions of this article will be made available by the authors, without undue reservation.

## Ethics statement

The studies involving human participants were reviewed and approved by Medical Ethics Committee of Fuyang People's Hospital. The patients/participants provided their written informed consent to participate in this study.

## Author contributions

HW, BW, and XR designed, conceived the study, and revised the manuscript. SW, XL, and XF enrolled patients, acquired the data, and performed the experiments. XW and MF analyzed the data, contributed to producing the charts, and drafted the manuscript. All authors contributed to the article and approved the submitted version.
