# Supplementary figures and images for: Safety and immunogenicity of inactivated COVID-19 vaccine CoronaVac and the RBD-dimer–based COVID-19 vaccine ZF2001 in chronic hepatitis B patients
Source: Front Med (Lausanne). 2023 Feb 8;10:1078666. doi: 10.3389/fmed.2023.1078666 (PMC9944390; doi:10.3389/fmed.2023.1078666)

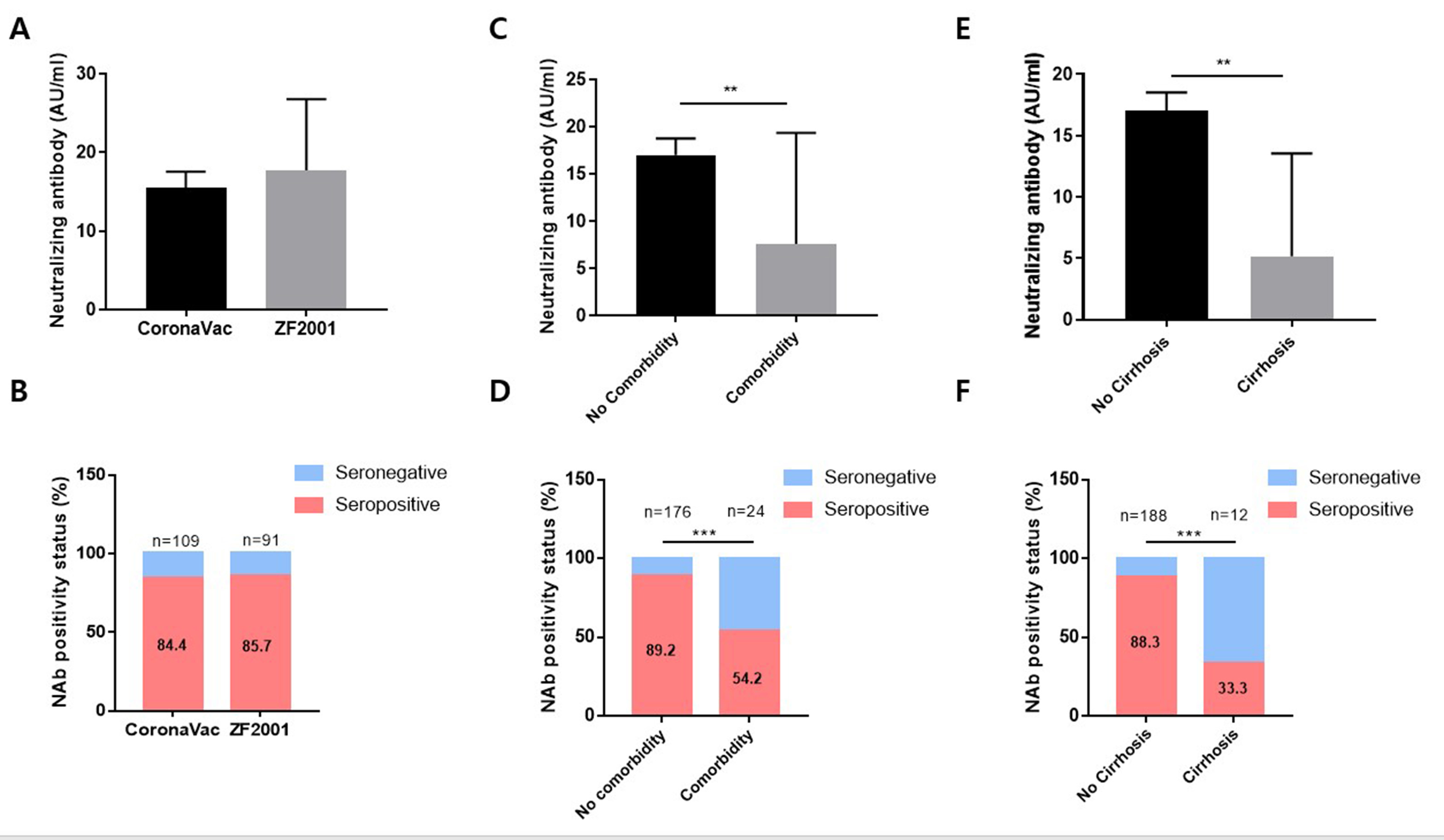

Supplement: Supplementary file 1 [file Image_1.JPEG]

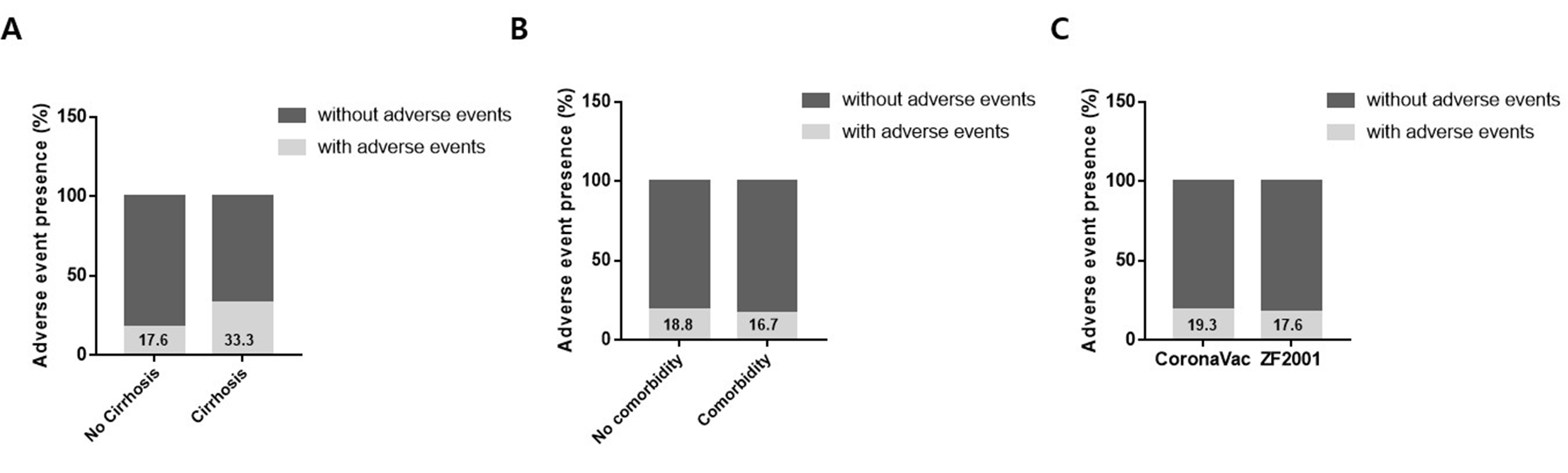

Supplement: Supplementary file 2 [file Image_2.JPEG]
